# Robust Dead Reckoning System for Mobile Robots Based on Particle Filter and Raw Range Scan

**DOI:** 10.3390/s140916532

**Published:** 2014-09-04

**Authors:** Zhuohua Duan, Zixing Cai, Huaqing Min

**Affiliations:** 1 Zhongshan Institute, University of Electronic Science and Technology of China, No. 713, Mingde Building, Zhongshan 528400, China; E-Mail: duanzhuohua@163.com; 2 School of Information Science and Engineering, Central South University, No. 204, Minzhu Building, Changsha 410083, China; E-Mail: zxcai@csu.edu.cn; 3 School of Software, South China University of Technology, Higher Education Mega Center, Guangzhou 510006, China

**Keywords:** mobile robots, fault diagnosis, robust dead reckoning, particle filters, raw scan matching

## Abstract

Robust dead reckoning is a complicated problem for wheeled mobile robots (WMRs), where the robots are faulty, such as the sticking of sensors or the slippage of wheels, for the discrete fault models and the continuous states have to be estimated simultaneously to reach a reliable fault diagnosis and accurate dead reckoning. Particle filters are one of the most promising approaches to handle hybrid system estimation problems, and they have also been widely used in many WMRs applications, such as pose tracking, SLAM, video tracking, fault identification, *etc.* In this paper, the readings of a laser range finder, which may be also interfered with by noises, are used to reach accurate dead reckoning. The main contribution is that a systematic method to implement fault diagnosis and dead reckoning in a particle filter framework concurrently is proposed. Firstly, the perception model of a laser range finder is given, where the raw scan may be faulty. Secondly, the kinematics of the normal model and different fault models for WMRs are given. Thirdly, the particle filter for fault diagnosis and dead reckoning is discussed. At last, experiments and analyses are reported to show the accuracy and efficiency of the presented method.

## 1. Introduction

Robust dead reckoning is a critical and challengeable issue for autonomous mobile robots in the presence of faults, such as the sticking of sensors, the slippage of wheels and the noisy readings of internal and/or external sensors. Sensor faults may change the kinematics and measurement models of wheeled mobile robots (WMRs). For example, the sticking of odometry sensors requires the system to switch the kinematics of WMRs. On the other hand, the faults of external sensors (such as a laser range finder, CCD camera, *etc.*) require a reliable perception model by fusing multiple external sensors.

When the WMRs are governed by faults, the accuracy of the dead reckoning system may decrease significantly. The difficulties of robust dead reckoning with faulty sensors include: (1) faults have to be identified accurately and quickly, for the kinematics and perception models are determined by the discrete fault models; (2) correct and accurate kinematics and perception models have to be built for different fault models; (3) in some cases, the dead reckoning can hardly be achieved when WMRs are seriously damaged.

Concerning the robust dead reckoning problem in the presence of various kinds of sensor faults, one of the intuitive methods includes a two-step process: firstly, fault models are identified with general fault diagnosis methods; secondly, robust dead reckoning is obtained according to different fault models. However, this methodology is generally time consuming and inaccurate.

In this paper, a particle filter-based method is proposed to diagnose faults and compute dead reckoning simultaneously. Particle filters have been widely used in fault diagnosis, pose tracking and simultaneously localization and mapping (SLAM) applications for WMRs. In this methodology, the discussed questions are modeled as a hybrid system estimation problem, where the discrete states of faults and the continuous states of dead reckoning are estimated simultaneously. An external sensor, namely a laser range finder, is used to correct the errors of internal sensors caused by faults or large noises. To achieve this purpose, a robust and fast raw scan projection method using polar coordinates is employed to evaluate the weights of particles. The main contribution of this paper is that it proposes a general method to handle the fault diagnosis of internal sensors and the accurate dead reckoning in the situation of sensor faults with a particle filter based on raw scan matching.

The remainder of the paper is organized as follows. In Section 2, we briefly reviewed the previous works on the relative topics of the robust dead reckoning methods of mobile robots, including fault diagnosis, robust perception, dead reckoning, *etc.* In Section 3, the general framework of the particle filter for the hybrid state estimation problem is put forward. In Section 4, the robust perception model for laser range finder is presented. In Section 5, fault diagnosis and robust dead reckoning based on the particle filter are discussed. The experimental results and analyses are given in Section 6.

## 2. Previous Works

### 2.1. Fault Diagnosis for Mobile Robots

Fault detection and diagnosis (FDD) is increasingly important for wheeled mobile robots (WMRs), especially those under unknown environments, such as planetary exploration [1]. Multiple model methods are widely used in sensor fault diagnosis for mobile robots [2–4]. In multiple models methods, the dynamics of each fault model are represented with Kalman filters (KFs). As the number of possible fault models increases exponentially over the number of components, the number of KFs increases respectively. Additionally, the precondition of KFs is that the process noise and the measurement noise are a zero mean Gaussian process with known covariance.

To deal with these problems, many researchers employ particle filters (PFs) to study fault diagnosis problems after the pioneering work of de Freitas [5]. However, general particle filters have the following drawbacks: degeneracy in sequential importance sampling (SIS), loss of diversity in sample importance resampling (SIR) [6] and the curse of dimensionality, *i.e.*, the rate of convergence of the approximation error decreases as the state dimension increases [7].

Many improvements in particle filters focus on tackling the problems mentioned above. For example, the Rao–Blackwellised particle filter (RBPF) decreases the sample number by only sampling the discrete states; the trade-off is that it must exploit linear-Gaussian models of each discrete state [5]; and the variable resolution particle filter (VRPF) tracks abstract states that may represent single state or sets of states [8]. Lookahead-RBPF takes account of the current readings of external sensors in the sampling step to improve the efficiency and accuracy [9].

Fox presented a statistical approach to increasing the efficiency of particle filters by adapting the size of sample sets, which bound the approximation error introduced by the sample-based representation of the particle filter, and the approximation error is measured by the Kullback–Leibler divergence (KLD-sampling) [10]. Duan presented an adaptive particle filter for soft fault compensation, which adapts the parameter of the noise variance of the process and the number of samples simultaneously [11].

Hashimoto *et al.* presented a method to diagnose the faults of internal and external faults of mobile robots. The gains of internal sensors (two encoders and one gyroscope) are estimated via the scan matching method of the laser range finder, which may be also influenced by abrupt faults. The laser range finder is supposed to be faulty if the laser images in several successive scans are not matched [12]. Another work conducted by Hashimoto *et al.* is a voting-based fault isolation approach, in which the velocity estimates with the four sensors (three LRSs and one dead reckoning) are compared with each other, and the sensor whose velocity estimates do not match the others is decided to be the faulty one [13].

Gage *et al.* put forward a survey on sensing assessment in unknown environments, especially for mobile robot applications [14]. They showed that only a few studies focused on the detection (and identification) of real exteroceptive sensor faults.

Recently, various kinds of data-driven methods have been employed to handle fault diagnosis for mobile robots when the system model can hardly be obtained. For example, Christensen *et al.* employed back-propagation neural network to synthesize fault detection components with a fault injection scheme [15]; Raphael *et al.* proposed an online data-driven fault detection for robot systems, which learns the posterior density based on online data collected with sensors [16]; Lau *et al.* put forward an adaptive data-driven error detection in swarm robotics with statistical classifiers, namely the receptor density algorithm (RDA), which is based on immune computation [17]; Lin *et al.* used a support vector machine (SVM) to handle fault diagnosis problem for mobile robots [18]. Most recently, model-based methods and data-driven methods have been integrated to handle fault diagnosis problem for UAVs, in which the model-based methods serves as a residual generator, and the data driven-methods are employed to detect an anomaly [19].

### 2.2. Robust Dead Reckoning for Mobile Robots

A dead reckoning system computes the relative transform between two consecutive frames of the robot, $A=\left[\begin{array}{cc} H & P\\ 0 & 1 \end{array}\right]$, where $H=\left[\begin{array}{cc} \text{cos}\theta & -\text{sin}\theta \\ \text{sin}\theta & \text{cos}\theta \end{array}\right]$ is a rotation matrix, and *P* =(*x*, *y*)*^T^* , is a translation vector.

The translation vector and rotation matrix are typically given by the readings of internal sensors, such as the gyroscope and encoders. However, these readings consist of both systematic and non-systematic errors. The former depend on the structure of the sensors and the mobile platform adopted; the latter are due to undesired interactions between the robot and the environment, such as slippage and sticking of wheels caused by uneven ground. Systematic errors, both deterministic and probabilistic, can be predicted. For example, the finite resolution of encoders causes a normally-distributed error [20]. Borenstein *et al.* presented a method for measuring and reducing systematic odometry errors of differential drive mobile robots, which is caused by uncertain wheelbase and unequal wheel diameters, based on carefully designed error models [20]. Non-systematic errors, which play a significant role in robust dead reckoning system, cannot be predicted. Meng *et al.* presented a trigonometry-based model to increase the accuracy of odometry-based pose estimation of a mobile robot with two steerable drive wheels. The underlying idea uses the ratio of two drive wheels' incremental displacements to detect non-systematic errors mainly caused by slippage [21]. It is suggested to take advantage of particular external sensors, such as the laser range finder and camera, to build an accurate environment map [20,22].

Ward *et al.* proposed a dynamic model-based wheel slip detector, which estimates longitudinal wheel slip and detects immobilized conditions of autonomous mobile robots operating on outdoor terrain [23]. Firstly, a tire traction/braking model is exploited to calculate vehicle dynamic forces with an extended Kalman filter framework. Internal sensors and GPS are then fused to estimate external forces and robot velocity. In this work, the GPS is used to estimate the velocity of the robot. However, the GPS is usually useless in indoor environments.

Chung *et al.* presented a method to improve dead reckoning accuracy with fiber optic gyroscopes (FOGs) in mobile robots [24]. The key idea is to build an accurate model for FOGs to handle the non-linearity of the scale factor and the temperature dependency of the gyroscope and fuse the sensor data from the FOG with the odometry system by an indirect Kalman filter. The accuracy of FOG is usually higher than that of encoders; however, the measurements of FOG itself will drift and cannot recover without external sensors.

Sekimori *et al.* proposed a dead reckoning method based on increments of the robot movements read directly from the floor using optical mouse sensors, in which the measurement values from multiple optical mouse sensors are compared to each other and only the reliable values are selected; accurate dead reckoning can be realized compared with the conventional method based on increments of wheel rotations [25]. This is a kind of hardware redundancy method, which is usually expensive and cannot be deployed in small robots, due to the limited volumes.

Cobos *et al.* put forward a method that combines a monocular vision system-based visual odometer and onboard odometer systems to reduce the errors of dead reckoning, which uses optical flow techniques and planar models to obtain qualitative 3D information and robot localization by using time integration series of acquired frames [26]. However, the accuracy of the monocular vision-based method is low, and the computational burden is high.

Up to now, almost all of the research concerning robust dead reckoning has focused on error estimation. Robust dead reckoning under the conditions of internal or external sensors being faulty is still an open problem. The difficulty of robust and accurate dead reckoning includes: (1) the sensor readings are noisy; and (2) an accurate model can hardly be obtained, especially under complicated environments and when components are faulty. External sensor readings can significantly improve the accuracy of dead reckoning; however, two key issues arise, *i.e.*, the perception model of the external sensor must be accurate, and the computational burden must be decreased to reach real-time estimation.

### 2.3. Raw Scan Matching

As mentioned above, the errors cannot be corrected inside the internal sensors of the dead reckoning system. A typical way to estimate accurate dead reckoning is to employ external sensors, such as a camera or a laser range finder. A laser range finder is more accurate and efficient than a camera, especially in indoor environments. In this section, we briefly review the scan matching methods for a laser range finder found in the current literature.

Scan matching approaches typically include two categories, namely feature-based matching [27,28] and point-to-point matching [29–33].

In feature-based matching, features, such as line segments, corners or range extrema, are extracted from laser scans and then matched. The difficulties for the feature-based method include: (1) features are difficult to extract in some situations, such as outdoor environments; (2) the corresponding feature matching is time consuming.

Typical point-to-point matching methods include iterative closet point (ICP) [32], iterative matching range point (IMRP) [33], iterative dual correspondence (IDC) [33], corresponding vector fitting sample and consensus (CVFSAC) [29], polar scan matching (PSM) [31] and inverse ray tracing (IVT) [34].

The point-to-point matching algorithms apply a so-called projection filter before matching. The computational complexity for the projection filter in the Euclidean coordinate system is typically *O*(*n*^2^), where n denotes the number of points. This is the case for the algorithms of ICP, IMRP, IDC and CVFSAC.

Another drawback of ICP-based methods is that they need an accurate initial guess. Minguez *et al.* defined a metric distance for ICP in the configuration space of the sensor, which takes into account both the translation and rotation error of the sensor [30]. However, how to choose an the parameter combining translation and orientation difference in the proposed metric is still a difficult issue [31]. Martinez *et al.* presented an GA-ICP scan matching method, which includes two steps. The genetic algorithm is used to search the optimal match roughly; after that, the ICP algorithm is employed to optimize the best guess of GA [35].

PSM works in the laser scanner's polar coordinate system, which takes advantage of the structure of the laser measurements and eliminates the need for an expensive search for corresponding points in other scan match approaches [31]. Similarly, Duan *et al.* presented a robust and fast measure for the estimation of assignment of two consecutive frames, which proposed a fast inverse ray tracing method [34]. Both PSM and IVT compute the projection in the polar coordinate system and reduce the computational complexity to *O*(*n*).

## 3. Hybrid System Estimation Based on Particle Filter

A particle filter is a Monte Carlo (*i.e.*, choosing randomly) method to monitor dynamic systems, which non-parametrically approximates a probabilistic distribution using weighted samples (particles). A particle filter gives a computationally feasible method for the state estimation of nonlinear and non-Gaussian hybrid systems. The term ‘hybrid’ denotes that the system states contain discrete and continuous parts. Fault diagnosis for a dynamic system is a typical kind of hybrid system, in which discrete states are fault modes and continuous states are determined according to the system to be estimated.

The main idea for using a particle filter as a hybrid system estimation method is described as follows. Let **S** represent the finite set of discrete models in the system; *s_t_* represent the discrete model of the system to be estimated at time *t* and *s_t_* ∈ **S**, {*s_t_*} represent discrete, first order Markov chain representing the evolution of the state over time; **x***_t_* stand for the multivariate continuous state of the system at time *t*. In Bayesian theory, the problem of hybrid system estimation consists of providing a belief (a distribution over the state set **S**) at each time step as it evolves according to the following transition model:

(1) $$p(s_{t}=j|s_{t-1}=i),i,j,\in \text{S}$$

Each of the discrete models changes the dynamics of the system. The non-linear conditional state transition models are denoted by *p*(**x***_t_*|**x***_t_*_−1_*,s_t_*). The state of the system is observed through a sequence of measurements, {**z***_t_*}, based on the perception model *p*(**z***_t_*|**x***_t_,s_t_*). The problem of hybrid state estimation consists of two consecutive steps. The first step is estimating the marginal distribution *p*(*s_t_*|**z**_1_*_..t_*) of the posterior distribution *p*(**x***_t_,s_t_*|**z**_1…_*_t_*). The second step is to estimate the continuous state distribution *p*(**x***_t_*|*s_t_*, **z**_1_…*_t_*) according the discrete models obtained previously.

A recursive estimate of this posterior distribution may be obtained using the Bayes filter:

(2) $$p({\text{x}}_{t},s_{t}|{\text{z}}_{1\ldots t})=\eta_{t}p({\text{z}}_{t}|{\text{x}}_{t},s_{t})\int \sum_{s_{t-1}}p({\text{x}}_{t},s_{t}|{\text{x}}_{t-1},s_{t-1})d{\text{x}}_{t-1}$$

There is no closed form solution for this recursion. Particle filters appropriate the posterior with a set of *N* fully instantiated state samples or particles $\left\{(s_{t}^{\left[1\right]},{\text{x}}_{t}^{\left[1\right]}),\cdots ,(s_{t}^{\left[N\right]},{\text{x}}_{t}^{\left[N\right]})\right\}$ and importance weights $\left\{w_{t}^{\left[i\right]}\right\}$:

(3) $${\hat{p}}_{N}({\text{x}}_{t},s_{t}|{\text{z}}_{1\cdots t})=\sum_{i=1}^{N}w_{t}^{\left[i\right]}\delta_{\left({\text{x}}_{t}^{\left[i\right]},s_{t}^{\left[i\right]}\right)}({\text{x}}_{t},s_{t})$$

where *δ*(.) denotes the Dirac delta function. The appropriation in Equation (3) approaches the true posterior density as *N* → ∞. Because it is difficult to draw samples from the true posterior, samples are drawn from a more tractable distribution *q*(.), which is called the proposal (or importance) distribution. The most widely used proposal distribution is the transition distribution (4):

(4) $$q\left(.\right)=p({\text{x}}_{t},s_{t}|{\text{x}}_{t-1},s_{t-1})$$

The importance weights are used to account for the discrepancy between the proposal distribution *q*(.) and the true distribution *p*(**x***_t_,s_t_*|**z**_1…_*_t_*). When the proposal distribution is given by Equation (4), the importance weight of sample $\left(s_{t}^{\left[i\right]},{\text{x}}_{t}^{\left[i\right]}\right)$ is:

(5) $$w_{t}^{\left[i\right]}=p({\text{z}}_{t}|{\text{x}}_{t}^{\left[i\right]},s_{t}^{\left[i\right]})$$

The general particle filter algorithm for hybrid system estimation is expressed in Algorithm 1.


**Algorithm 1:** General particle filter for hybrid system estimation.
Initialize: For *N* particles 
${\left\{{\text{x}}_{t}^{\left[i\right]},s_{t}^{\left[i\right]}\right\}}_{i=1}^{N}$, sample discrete state 
${\left\{s_{0}^{\left[i\right]}\right\}}_{i=1}^{N}$, from the prior *p*(*s*_0_), sample 
${\left\{{\text{x}}_{t}^{\left[i\right]}\right\}}_{i=1}^{N}$ from 
$p({\text{x}}_{0}|s_{0}^{i})$.;**for**
*each time step t*
**do** **for**
*each particle i*
**do**  Sample discrete state: 
$s_{t}^{\left[i\right]}\sim p(s_{t}|s_{t-1}^{\left[i\right]})$;  Sample continuous state: 
${\text{x}}_{t}^{\left[i\right]}\sim p({\text{x}}_{t}|{\text{x}}_{t-1}^{\left[i\right]},s_{t}^{\left[i\right]})$;  Update 
$w_{t}^{\left[i\right]}=p(z_{t}|{\text{x}}_{t}^{\left[i\right]},s_{t}^{\left[i\right]})$; **end** Discrete state estimation: *ŝ_t_* = *argmax_st_p̂_N_*(*s_t_*|**z**_1.._*_t_*); Continuous state estimation: 
${\hat{\text{x}}}_{t}=\sum_{i=1,s_{t}^{\left[i\right]}={\hat{s}}_{t}}^{N}w_{t}^{\left[i\right]}{\text{x}}_{t}^{\left[i\right]}$; Normalize the weights: 
$w_{t}^{\left[i\right]}=\frac{w_{t}^{\left[i\right]}}{\sum_{N}^{i=1}w_{t}^{\left[i\right]}}$; Resample: generating 
${\left\{{\text{x}}_{t}^{\left[i*\right]},s_{t}^{\left[i*\right]}\right\}}_{i=1}^{N}$, such that 
$p(({\text{x}}_{t}^{\left[i*\right]},s_{t}^{\left[i*\right]})=({\text{x}}_{t}^{\left[j\right]},s_{t}^{j}))=w_{t}^{\left[j\right]}$, 
${\left\{{\text{x}}_{t}^{\left[i\right]},s_{t}^{\left[i\right]}\right\}}_{i=1}^{N}={\left\{{\text{x}}_{t}^{\left[i*\right]},s_{t}^{\left[i*\right]}\right\}}_{i=1}^{N},w_{t}^{\left[i\right]}=1/N$;**end**


## 4. Perception Model of Laser Range Finder Based on Scan Projection in Polar Coordinates

In this section, we construct a perception model for a laser range finder based on the scan projection method in polar coordinates. This kind of scan projection was firstly proposed in [31]. We first filter out noisy rays with a kind of segmentation technique. Then, we employ the scan projection method of PSM to quickly compute corresponding rays of two consecutive scans. The distances of corresponding points, after transforming to the same frame, are influenced by two factors: the accuracy of relative transformation and the changes of the environment.

### 4.1. Segment Analysis and Effective Scan Window

Let *R_t_* denote range data at time *t*,

(6) $$R_{t}=\{b_{t}^{j}=(\rho_{t}^{j},\alpha_{t}^{j})\},j\in [1..L]$$

where $b_{t}^{j}$ is the *j* − *th* ray of the scan *R_t_*, *L* is the number of rays of the scan, $\rho_{t}^{j}$ is the range readings of the *j* − *th* ray and $\alpha_{t}^{j}$ is the orientation of the *j* − *th* ray, which is calculated as follows,

(7) $$\alpha_{t}^{j}=\varrho +(j-1)\varsigma$$

where *ϱ* is the angular offset of the first ray and *ς* is the angular resolution.

For example, in typical scan readings of LMS291, *L* = 361, *ϱ* =0 and *ς* =0.5*deg*. We will show later that the angular structure of scan may play an important role in the proposed perception model.

The process of segment analysis of a raw scan is to divide the rays into several segments; each segment of scan is a set of consecutive rays in the scan, such that the range of consecutive rays that does not jump dramatically. Segment analysis of scan *R_t_* is done by dividing it into segments as follows,

(8) $$R_{t}=\overset{c_{t}}{\underset{k=1}{\cup }}G_{t}^{k}$$

where $G_{t}^{k}=\{b_{t}^{i}|i\in [{\text{start}}_{t}^{k}..{\text{end}}_{t}^{k}]\}$ denotes the *k* − *th* segment, *c_t_* denotes the number of segments of the scan, ${\text{start}}_{t}^{1}=1,{\text{end}}_{t}^{c_{t}}=L,{\text{start}}_{t}^{k+1}={\text{end}}_{t}^{k}+1,|\rho_{t}^{i+1}-\rho_{t}^{i}|<\text{gap},i+1,i\in [{\text{start}}_{t}^{k}..{\text{end}}_{t}^{k}],∥\rho_{t}^{{\text{start}}_{t}^{k+1}}-\rho_{t}^{{\text{end}}_{t}^{k}}|\geq \text{gap},\text{gap}$ denotes a threshold.

Let ${\text{num}}_{t}^{k}={\text{end}}_{t}^{k}-{\text{start}}_{t}^{k}+1$ denote the numbers of rays in segment *k*, and ${\tilde{\rho }}_{t}^{k}=\frac{1}{{\text{num}}_{t}^{k}}\sum_{i={\text{start}}_{t}^{k}}^{{\text{end}}_{t}^{k}}\rho_{t}^{i}$ denotes the average distance of segment *k*. ${\text{num}}_{t}^{k}$ and ${\tilde{\rho }}_{t}^{k}$ are important features about segments.

Based on the features of the segments of raw scan, noise readings can be filtered out with the following method to construct the effective scan windows for the readings,

(9) $$W_{t}=\overset{c_{t}}{\underset{k=1}{\cup }}G_{t}^{k},{\text{num}}_{t}^{k}>\gamma ,\lambda_{2}\geq {\tilde{\rho }}_{t}^{k}\geq \lambda_{1}$$

where *W_t_* denotes the effective scan of *R_t_*, and *γ*, λ_2_, λ_1_ denote the threshold, respectively. λ_2_ depends on the maximal perception distance of the range finder. λ_1_ stands for the minimal valid measurements of environments. This is because the range finder is mounted on the robot, and for a mechanical reason, the minimal distance from the range finder to the environment is larger than zero. For the LMS291, we set *γ* =5, λ_2_ = 81.9 *m* and λ_1_ =0.03 *m*.

### 4.2. Scan Projection in Polar Coordinates

Let Γ*_t_* denote the coordinate frame of scanner at time *t*, and the homogeneous transformation matrix of Γ*_t_* with respect to Γ*_t_*_−1_ is *A_t_*,

(10) $$A_{t}=\left[\begin{array}{cc} H_{t} & P_{t}\\ 0 & 1 \end{array}\right]$$

where $H_{t}\left[\begin{array}{cc} \text{cos}\theta_{t} & -\text{sin}\theta_{t}\\ \text{sin}\theta_{t} & \text{cos}\theta_{t} \end{array}\right]$ is a rotation matrix, and *P_t_* = (*x_t_, y_t_*)*^T^*, is a translation vector. Both the rotation matrix and translation vector are determined by the continuous state of the hybrid system, namely **x***_t_*,

(11) $${\text{x}}_{t}={\left(x_{t},y_{t},\theta_{t}\right)}^{T}$$

The purpose of the scan projection is to find out what the current scan would look like if it were taken from the reference position given the pose estimation **x***_t_*. The fast scan projection of PSM contains the following steps.

Step 1: Compute the projected ray of each ray $b_{t}^{i}$ of the current scan, and let $d_{t}^{i}$ and $\gamma_{t}^{i}$ denote the length and orientation of the projected ray, respectively.

(12) $$d_{t}^{i}=\sqrt{{\left(\rho_{t}^{i}\text{cos}(\theta_{t}+\alpha_{t}^{i})+x_{t}\right)}^{2}+{\left(\rho_{t}^{i}\text{sin}(\theta_{t}+\alpha_{t}^{i})+y_{t}\right)}^{2}}$$

(13) $$\gamma_{t}^{i}=\text{atan}2(\rho_{t}^{i}\text{sin}(\theta_{t}+\alpha_{t}^{i})+y_{t},\rho_{t}^{i}\text{cos}(\theta_{t}+\alpha_{t}^{i})+x_{t})$$

Step 2: Calculate the expectation rays that would have been measured between $\gamma_{t}^{i-1}$ and $\gamma_{t}^{i}$ with interpolation, where $\left(d_{t}^{i-1},\gamma_{t}^{i-1}\right)$ and $\left(d_{t}^{i},\gamma_{t}^{i}\right)$ denote two consecutive projected measurements from the same segments.

Let $j_{1}=\text{floor}(\frac{\gamma_{t}^{i}-\rho }{\varsigma }),\;j_{0}=\text{ceil}(\frac{\gamma_{t}^{i-1}-\rho }{\varsigma })$. For every *j*(*j* > = *j*_0_ and *j* < = *j*_1_), compute the expected distance of the ray *j* of scan *R_t_*_−1_ with interpolation as follows,

(14) $$d(j,R_{t-1})=\frac{d_{t}^{i}-d_{t}^{i-1}}{\gamma_{t}^{i}-\gamma_{t}^{i-1}}(\alpha_{t-1}^{j}-\gamma_{t}^{i-1})+d_{t}^{i-1}$$

The discrepancy of the expectation and measurement of the ray $b_{t-1}^{j}$ is,

(15) $$e_{t}^{j}=d(j,R_{t-1})-\rho_{t-1}^{j}$$

### 4.3. Robust Perception Model

The robust perception model calculates *p*(*R_t_*|*R_t_*_−1_, **x***_t_*). Notice that the error $e_{t}^{i}$ is influenced by the following aspects (1) the accuracy of continuous state of **x***_t_*; (2) dynamic environments, such as occlusion by dynamic objects; (3) the abnormality of the laser range finder, such as separated beams.

A robust perception model is sensitive to the accuracy of **x***_t_* and robust to dynamic environments and the abnormality of the readings of sensors.

Let the measurement noise of a laser range finder follow a normal distribution with a variance of *σ*^2^.

For the laser scanner SICK-LMS291, σ is set as 10 mm. The likelihood $p(b_{t-1}^{j}|R_{t-1},{\text{x}}_{t})$ is,

(16) $$p(b_{t-1}^{j}|R_{t-1},{\text{x}}_{t})=\frac{1}{\sqrt{2\pi \sigma }}e^{-\frac{e_{t}^{j}}{2\sigma^{2}}}$$

Our perception model computes the likelihood with Equation (17).

(17) $$p(R_{t}|R_{t-1},{\text{x}}_{t})=\frac{1}{\left|R_{t-1}\right|}\sum_{b_{t-1}^{j}\in R_{t-1}}p(b_{t-1}^{j}|R_{t-1},{\text{x}}_{t})$$

## 5. Fault Diagnosis and Robust Dead Reckoning Based on Particle Filter

In this section, we give the detailed ideas of our work on the problem of fault diagnosis and robust dead reckoning based on a particle filter and raw scan matching. Firstly, the fault models, as well as the correspondent kinematics of different fault models are derived for a wheeled mobile robots equipped with two encoders, one fiber gyroscope and one laser range finder. Secondly, a fast and robust raw scan matching method, which is called ‘inverse ray tracing’, is discussed. In the presented method, the ‘inverse ray tracing’ method served as the perception model, which may be used to weight the particles. At last, the complete algorithm is presented.

### 5.1. Fault Models and Kinematics

For the problem of fault diagnosis and a dead reckoning system based on particle filters, the fault models and there kinematics are described as follows. Four kinds of discrete models are taken into consideration, as shown in Table 1.

Each discrete fault model determines the continuous state transition. In robust dead reckoning, the continuous state is **x***_t_* = (*x_t_*, *y_t_*, *θ_t_*)*^T^*. In the model state, the kinematics model is shown as Equation (18).

(18) $$\{\begin{array}{ll} \theta_{t} & =\tau_{t}\cdot {\dot{\theta }}_{t}\\ x_{t} & =\tau_{t}\cdot \upsilon_{t}\cdot \text{cos}(\theta_{t})\\ y_{t} & =\tau_{t}\cdot \upsilon_{t}\cdot \text{sin}(\theta_{t}) \end{array}$$

where *υ_t_* and *θ̇_t_* denote the linear speed and yaw rate of the robot at time step *t*, which are recorded with encoders and the gyroscope, respectively. Normally, *υ_t_* and *θ̇_t_* are reckoned based on the readings of the internal sensors, namely, encoders and the gyroscope. *τ_t_* denotes the interval of time step *t*.

Let $e_{t}^{L},e_{t}^{R}$ denote the linear velocity of left wheel and right wheel, which are obtained from the readings of the encoders by multiplying the radius of the wheels; *g_t_* denotes the yaw rate, which is the readings of the gyroscope, respectively. The velocity kinematics model of the normal state (model 1) is as follows,

(19) $$\{\begin{array}{ll} \upsilon_{t}^{1} & =(e_{t}^{L}+e_{t}^{R})/2\\ {\dot{\theta }}_{t}^{1} & =g_{t} \end{array}$$

Notice that the yaw rate can be measured with the gyroscope and reckoned with the measurements of encoders as follows,

(20) $${\dot{\theta }}_{t}=(e_{t}^{R}-e_{t}^{L})/D$$

where *D* denotes the axis length.

The gyroscope is more accurate than the encoder. In a normal situation, we use the readings of the gyroscope as the estimation of the yaw rate. When the gyroscope is stuck, we use the readings of the encoders to reckon the yaw rate according to Equation (20). Similarly, the readings of one encoder can be computed based on the readings of the other encoder and the gyroscope, according to Equation (20). In this way, we get the velocity kinematics for the fault Models 2, 3 and 4.

The velocity kinematics for fault Mode 2 is as follows,

(21) $$\{\begin{array}{ll} \upsilon_{t}^{2} & =e_{t}^{R}-D\cdot g_{t}/2\\ {\dot{\theta }}_{t}^{2} & =g_{t} \end{array}$$

The velocity kinematics for fault Mode 3 is as follows,

(22) $$\{\begin{array}{ll} \upsilon_{t}^{3} & =e_{t}^{L}+D\cdot g_{t}/2\\ {\dot{\theta }}_{t}^{3} & =g_{t} \end{array}$$

The velocity kinematics for fault Mode 4 is as follows,

(23) $$\{\begin{array}{ll} \upsilon_{t}^{4} & =(e_{t}^{L}+e_{t}^{R})/2\\ {\dot{\theta }}_{t}^{4} & =(e_{t}^{R}-e_{t}^{L})/D \end{array}$$

### 5.2. Particle Filter for Fault Diagnosis and Dead Reckoning

In this paper, robust dead reckoning is defined as computing the relative transform between two consecutive frames based on the readings of internal sensors and raw range data matching of two consecutive frames. The initial guess is based on the readings of internal sensors and the fault models. The particles are then drawn and evaluated according to the matching accuracy of consecutive raw data of laser range finder.

The particle filter for simultaneous fault diagnosis and robust dead reckoning is shown as Algorithm 2. The importance sampling is shown from Step 3 to Step 13. Firstly, during Step 3–9, the loop guarantees that every fault model is sampled by at least one particle. Noticed that Step 8 computes the weights of the dead reckoning of different models. Step 11 shows that the discrete particles are drawn according to the weights obtained with Step 8.

## 6. Experimental Results

### 6.1. The Robot and Experimental Scenario

**Figure 1.**
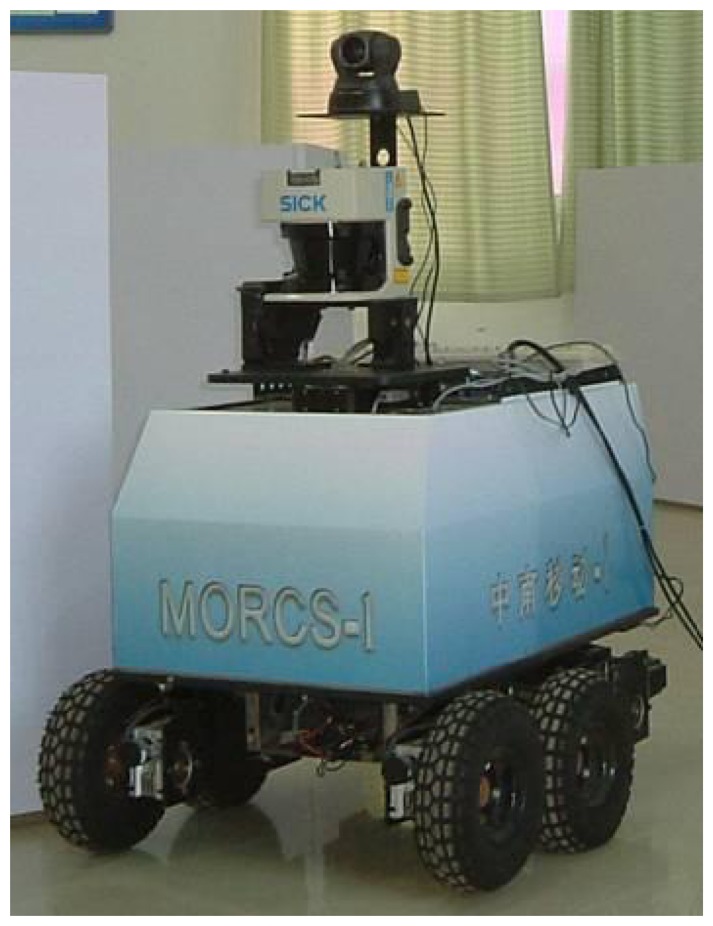
The robot MORCS-1.

**Figure 2.**
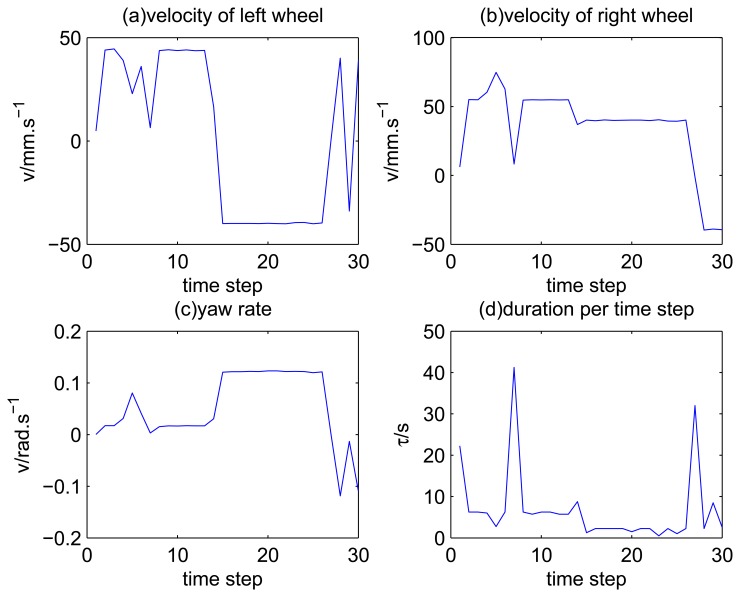
Velocity obtained from the readings of internal sensors.

The experimental data are obtained with the robot, MORCS-1, as shown in Figure 1, which is driven by a person remotely through a narrow door. The speed is about 50 mm/s. The axis length *D* is about 0.6 m. The MORCS-1 is equip with four encoders, one gyroscope one laser range finder (LMS291) and one CCD camera. LMS291 is a 2D laser range finder produced by SICK. LMS291 has a scanning range up to 81.92 meters from negative 90 degrees to 90 degrees at every 0.5 degree. The range resolution of LMS291 is 10 mm [36]. For convenience, the sampling time of the odometry and laser scanner are both set as 0.25 s. In our experimental scenarios, we only use the data of two encoders, the gyroscope and the laser range finder. The velocity obtained from the internal sensors is shown in Figure 2. Figure 2a,b shows the mean speed during the time interval *τ_t_* of the left wheel and the right wheel, respectively. Figure 2c shows the mean angular speed of the robot. Figure 2d shows the value of each time step.


**Algorithm 2:** Particle filter for simultaneous fault diagnosis and robust dead reckoning.
Initialize: Set particle number *N*, *S* = {1, 2, 3, 4}.;**for**
*each time step t*
**do** Determine the time interval *τ_t_*, such that, the |*W_t_*| > *reliabeScanTh*, the linear and angular distance of the robot is less than *distotalth* and *angtotalth*, respectively.; **for**
*i=1*
**to**
*4*
**do**  
$s_{t}^{\left[i\right]}=i$;  
$\theta_{t}^{\left[i\right]}=\tau_{t}\cdot {\dot{\theta }}_{t}^{i}$;  
$x_{t}^{\left[i\right]}=\tau_{t}\cdot \upsilon_{t}^{i}\cdot \text{cos}(\theta_{t}^{\left[i\right]})$;  
$y_{t}^{\left[i\right]}=\tau_{t}\cdot \upsilon_{t}^{i}\cdot \text{sin}(\theta_{t}^{\left[i\right]})$;  compute weight, 
$w_{t}^{\left[i\right]}=p(R_{t}|R_{t-1},{\text{x}}_{t}^{\left[i\right]})$, according to Equation (17); **end** **for**
*i=5*
**to**
*N*
**do**  Draw 
$s_{t}^{\left[i\right]}$, st ,
$s_{t}^{\left[i\right]}=j\propto w_{t}^{\left[j\right]}$, *j* ∈ [1..4];  Draw continuous state: 
${\text{x}}_{t}^{\left[i\right]}\sim p({\text{x}}_{t}|{\text{x}}_{t-1}^{i},s_{t}^{\left[i\right]})$ according to kinematic model of different fault modes, which is given by Equations (19), (21), (22) and (23), and the variance of state noise is governed by 
$w_{t}^{\left[s_{t}^{\left[i\right]}\right]}$.;  Compute weights: 
$w_{t}^{\left[i\right]}=p(R_{t}|R_{t-1},{\text{x}}_{t}^{\left[i\right]})$, according to Equation (17); **end** Calculate marginal probability distribution 
${\hat{p}}_{N}(s_{t}|{\text{z}}_{1..t})=\sum_{i=1}^{N}w_{t}\delta_{s_{t}^{\left[i\right]}}(s_{t})$; Fault diagnosis: state estimation 
${\hat{s}}_{t}^{\text{MAP}}={\text{argamax}}_{s_{t}}{\hat{p}}_{N}(s_{t}|{\text{z}}_{1..t})$; Dead reckoning estimation: 
${\hat{\text{x}}}_{t}={\text{argamax}}_{{\text{x}}_{t}^{\left[i\right]}}w_{t}^{\left[i\right]}$;**end**


**Figure 3.**
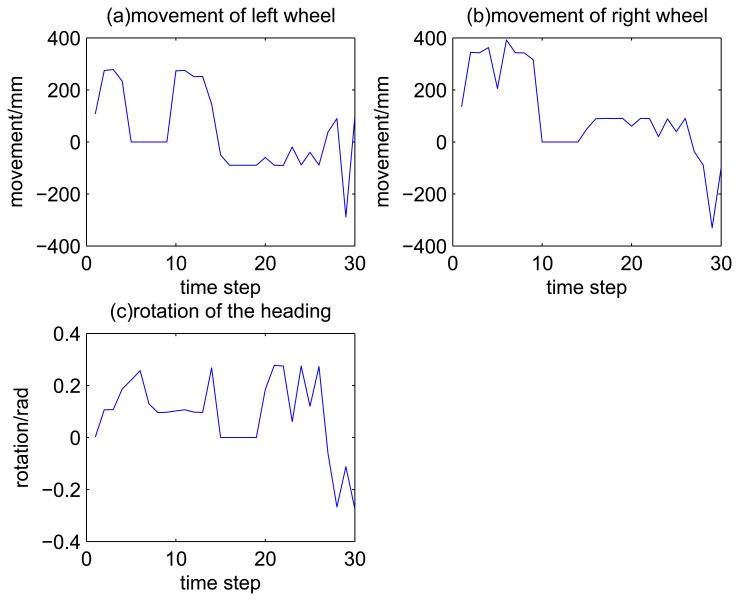
Movement of the mobile robot for each time step with fault injection.

The movement of the robot at each time step is shown in Figure 3. The faults are simulated by setting the value of movement of some time step to zero. Specifically, during time Steps 5 to 9, the movement of the left wheel is set as zero (set the fault model as 2). During time Steps 10 to 14, the movement of the right wheel is set as zero (set the fault model as 3). During time Steps 15 to 19, the angular movement is set as zero (set the fault model as 4).

The results are obtained off line with MATLAB. The particle number *N* is set as 200. The distance threshold between two time step, *distotalth*, is set as 300 mm, and the angle threshold between two time step, *angtotalth*, is set as 15 degrees. The threshold *reliabeScanT h*, which controls the reliability of raw scan readings, is set as 240.

### 6.2. The Result of Fault Diagnosis

**Figure 4.**
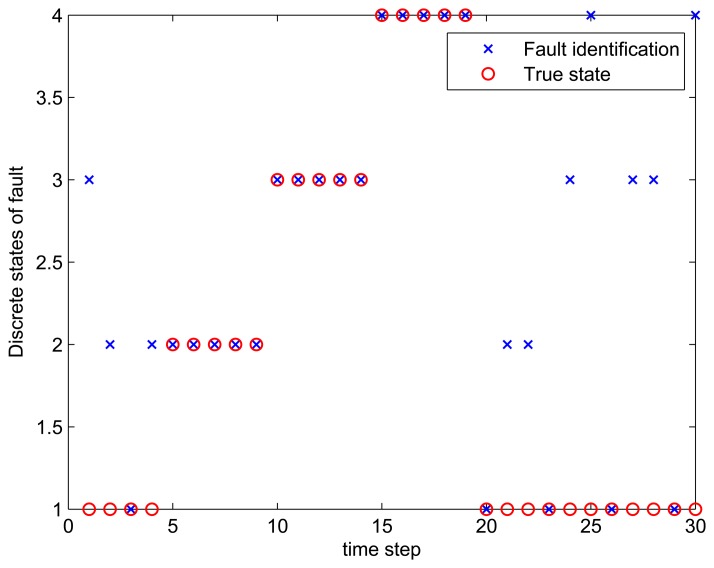
Fault diagnosis results.

The result of fault diagnosis is shown in Figure 4 and Table 2.

Figure 4 shows the results of fault diagnosis, as well as the true states. It shows that the algorithm works well during the periods of time Steps 5 to 19, when one internal sensor is stuck at zero. There are 10-times the misdiagnoses during the whole testing data of 30 time steps. Most misdiagnoses occur in the situation of ‘no faults’. This is mainly because of the redundancy of the system, *i.e.*, four kinds of kinematics are all correct when the system is normal, so the likelihoods of all models are nearly the same. This kind of diagnosability will be discussed in the next subsection.

Table 2 shows the likelihood of four kind of dead reckoning estimation of different fault models of the first 20 time steps. It shows that during the period of time Steps 5 to 9, the score of Model 2 is significantly larger than the scores of other models, this is consistent with fault Model 2 injected at this period. Similarly, during time Steps 10 to 14, the score of Model 3 is dramatically larger than the score of others; during time Steps 15 to 19, the score of Model 4 is larger the score of others, obviously.

### 6.3. Accuracy of the Perception Model

**Figure 5.**
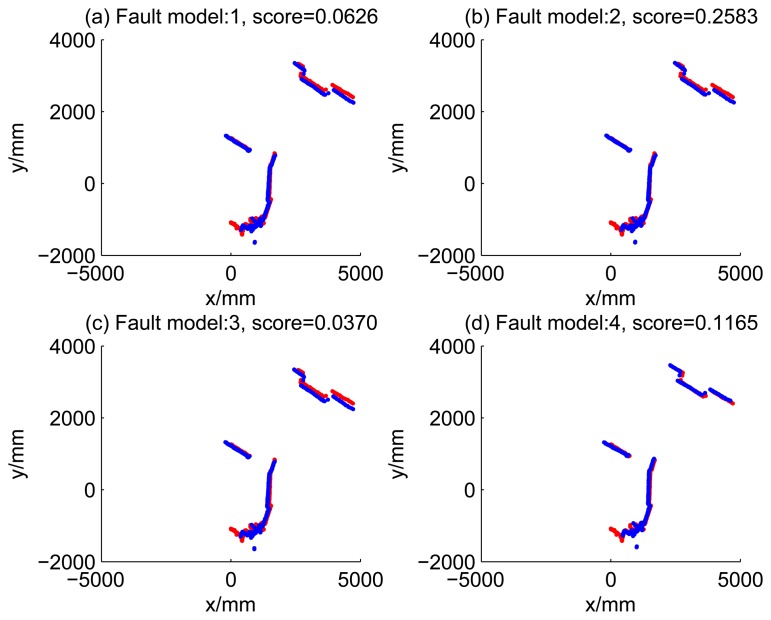
Raw scan match of the initial guess of four fault models (time Step 5).

**Figure 6.**
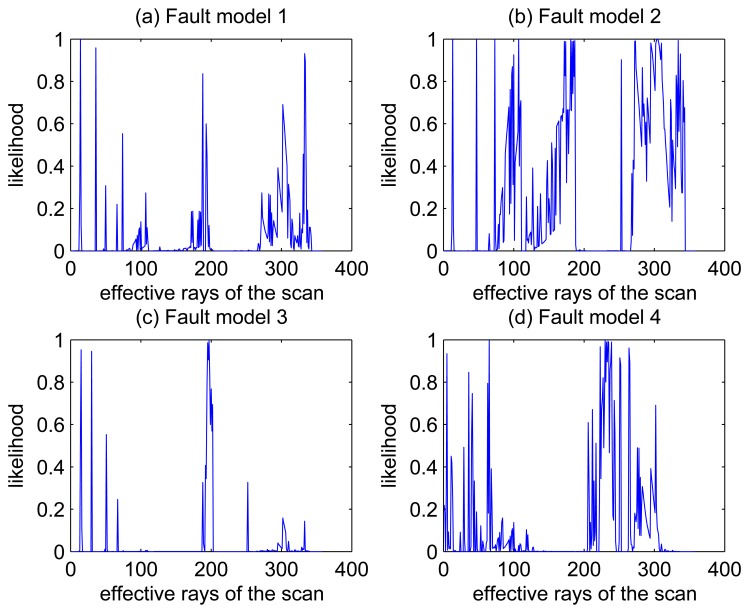
Likelihood of rays of the initial guess of four fault models (time Step 5).

**Figure 7.**
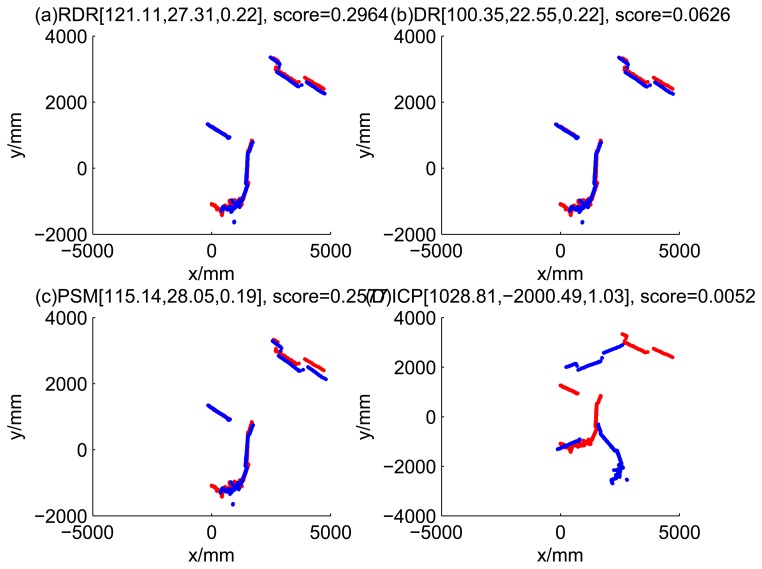
Raw scan match of the results of four methods (time Step 5).

**Figure 8.**
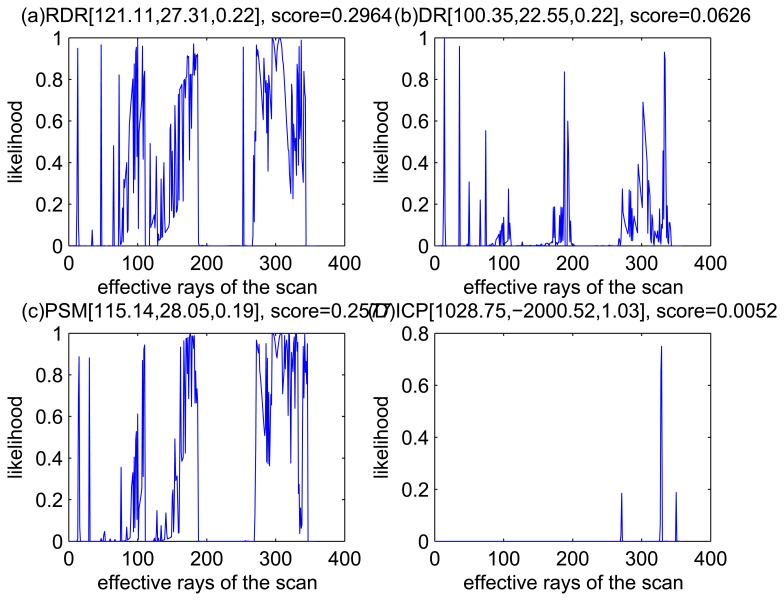
Likelihood of the rays of results of four methods (time Step 5).

Figures 5, 6, 7 and 8 illustrate the accuracy of the perception model. Figure 5 illustrates the raw scan match of the initial guess of four fault models at time Step 5. The score of fault Model 2 is the largest, and the matching result is also the best among the four subfigures. Accordingly, Figure 6 shows the likelihood of rays of the initial guess of four fault models (time Step 5). It shows that there are more high likelihood rays in the second model (subfigure (b)) than others. Figures 7 and 8 illustrate the match result of four method at time Step 5. It show that the match results and the likelihoods are consistent.

### 6.4. Accuracy of Robust Dead Reckoning

The accuracy of the proposed robust dead reckoning method is discussed in this section. Four kinds of method are implemented. The first one is the robust dead reckoning method (RDR for short) proposed in this paper. The second is the conventional dead reckoning method, which uses the readings for internal sensors directly (DR for short). The third is the PSM method. The fourth is the GA-ICP method (ICP for short). The GA is implemented with the sampling step of our method, but we only use the particles of normal models. ICP is then performed based on the best particle of normal models.

**Figure 9.**
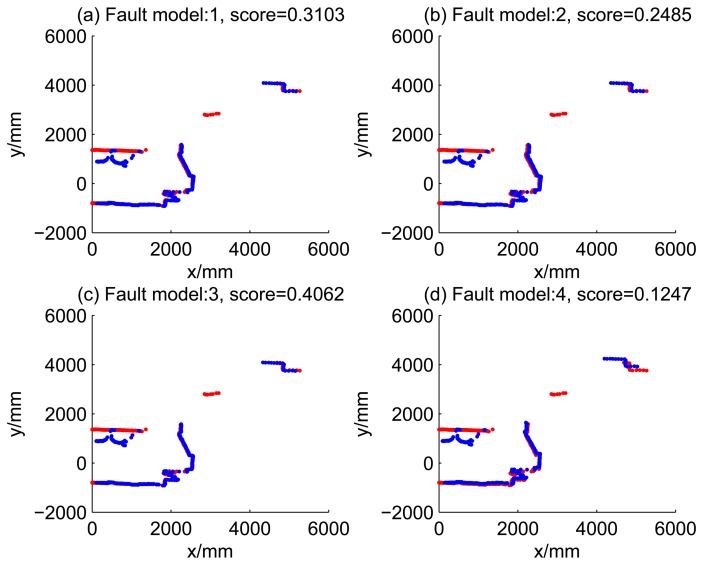
Case 1a: No faults, fault model = 1 (time Step 1).

**Figure 10.**
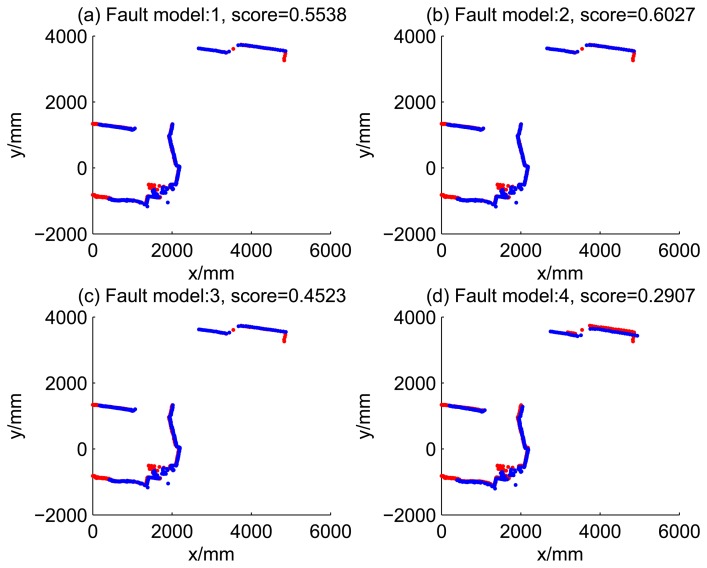
Case 1b: No faults, fault model = 1 (time Step 3).

**Figure 11.**
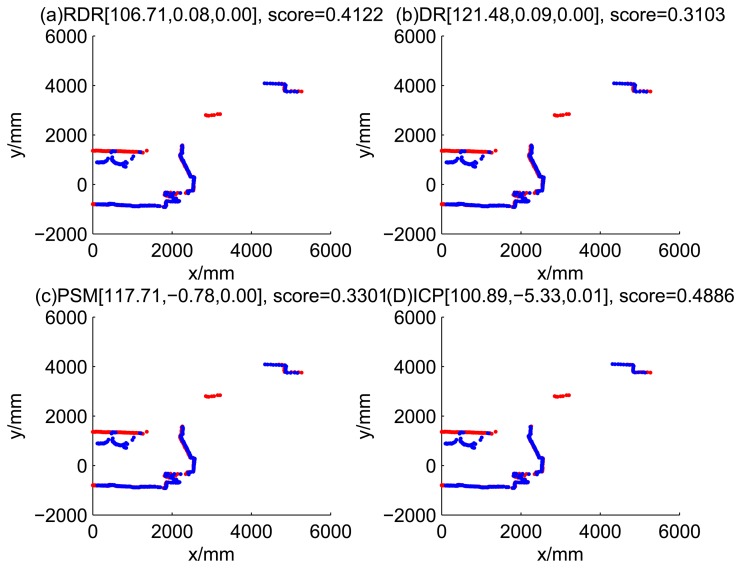
Robust dead reckoning result for Case 1a (time Step 1).

**Figure 12.**
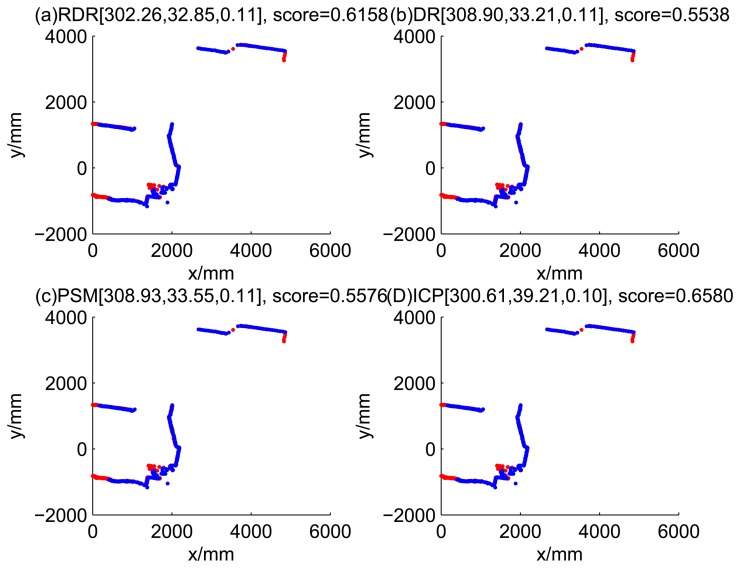
Robust dead reckoning result for Case 1a (time Step 3).

Figures 9, 10, 11 and 12 show the raw ray matching results of four kinds of dead reckoning (based on different fault models) and robust dead reckoning, in the situation of fault Model 1, *i.e.*, the case of ‘no fault’. It is shown that the four kinds of dead reckoning are almost the same.

Similarly, Figures 13, 14, 15 and 16 show the matching results of the case of fault Model 2, *i.e.*, ‘left encoder error’. Figures 17, 18, 19 and 20 show the results of the case of fault Model 3. Figures 21, 22, 23 and 24 show the results of the case of fault Model 4.

These figures show that in the case of ‘no faults’, the ICP method is superior to others. The proposed RDR method is superior to others in most cases when one of the internal sensors is stuck at zero. This is mainly because the ICP and PSM method do not take into consideration the faults. They suppose that the robot is in the normal state and then employ a bad initial guess. In fact, one can improve the accuracy of dead reckoning by taking the results of RDR as an initial guess of the PSM or ICP methods.

### 6.5. Diagnosability

There are three typical cases that are hard to diagnose. The first is that the kinematics of different models has a similar performance. The second is the that real state has not been modeled in the system. The third is that the readings of the external sensors are seriously damaged.

**Figure 25.**
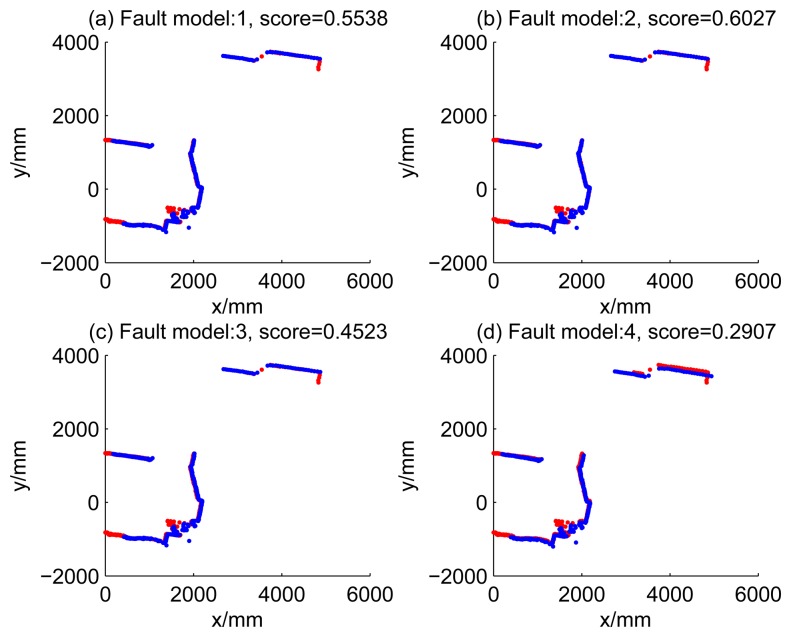
Diagnosability: No faults.

**Figure 26.**
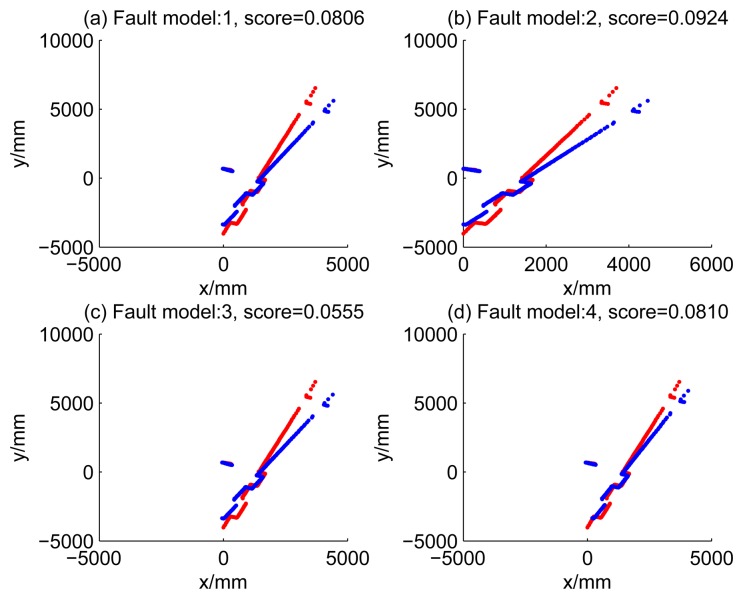
Two sensors are stuck.

Figure 25 shows the case of ‘no faults’; in this case, each model is correct, because of redundancy between the gyroscope and encoders. Therefore, every model has similar weights. Figure 26 shows the case of ‘two faults’, that is to say, two sensors are faulty simultaneously. Since none of the models given by the system can govern the situation, the weights of all four models are insignificant.

The presented robust dead reckoning system uses the raw scan of the laser range finder to improve the accuracy of dead reckoning. If the internal and external sensors both have faults in a short time interval (and there are no other sensors for help), the problem can be modeled as a kidnap problem, and some kind of global positioning method is recommended to handle this kind of situation.

## 7. Conclusion

In this paper, a robust accurate dead reckoning method based on particle filters is put forward by integrating the fault diagnosis of internal sensors and the readings of a laser range finder. In this framework, fault diagnosis and dead reckoning is computed simultaneously. The discrete fault models govern the continuous state transition of the dead reckoning system. The errors are corrected with the reading of external sensor, *i.e.*, laser range finder. The perception model of external sensors is fast and accurate by exploring the inverse ray tracing method. Experimental results and analysis are given. The presented method can serve as the first step for many other applications, such as SLAM, robust navigation, exploration, object tracking, and so on.

## Figures and Tables

**Figure 13.**
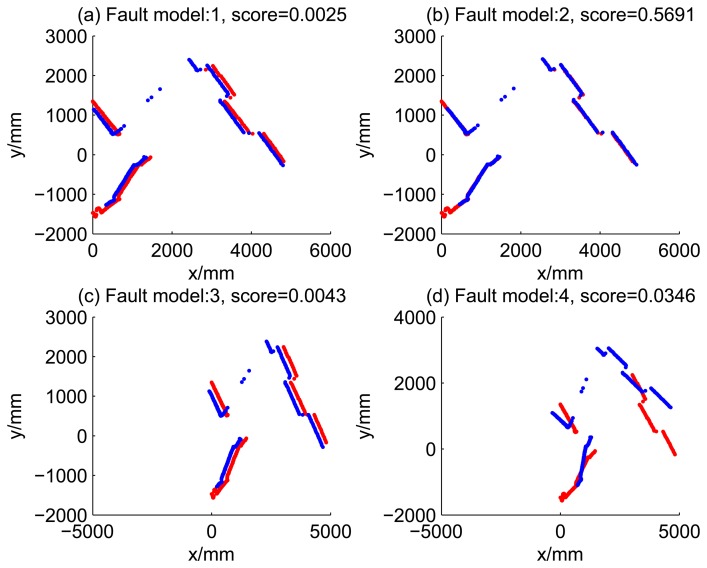
Case 2a: Left encoder stuck, fault model = 2 (time Step 7).

**Figure 14.**
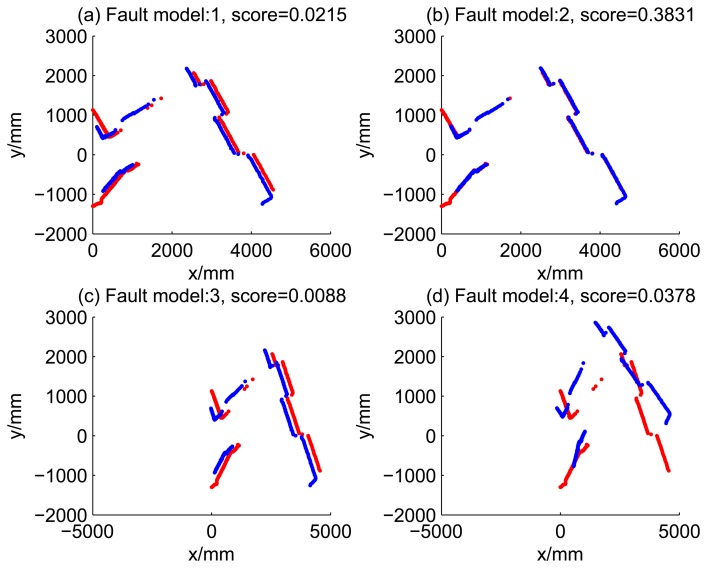
Case 2b: Left encoder stuck, fault model = 2 (time Step 8).

**Figure 15.**
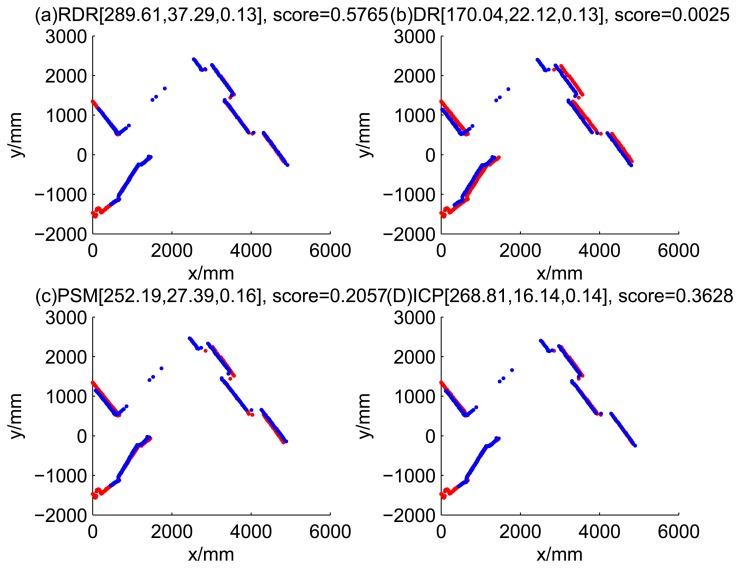
Robust dead reckoning result for Case 2a (time Step 7).

**Figure 16.**
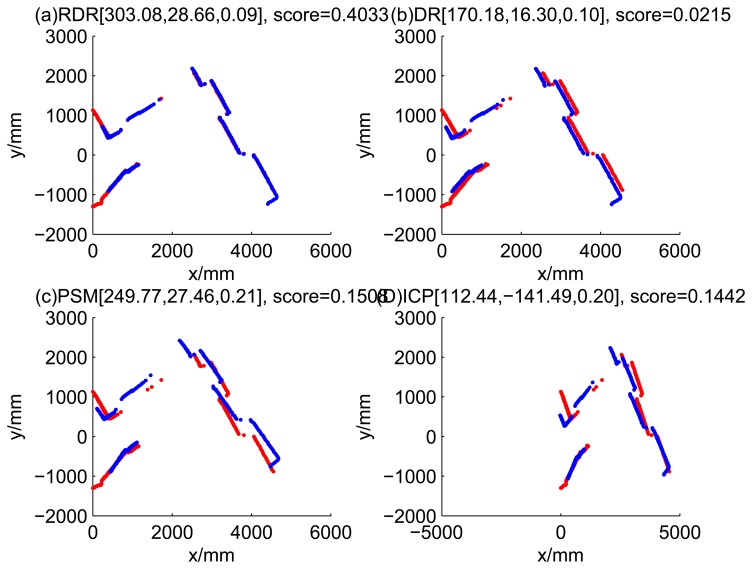
Robust dead reckoning result for Case 2b (time Step 8).

**Figure 17.**
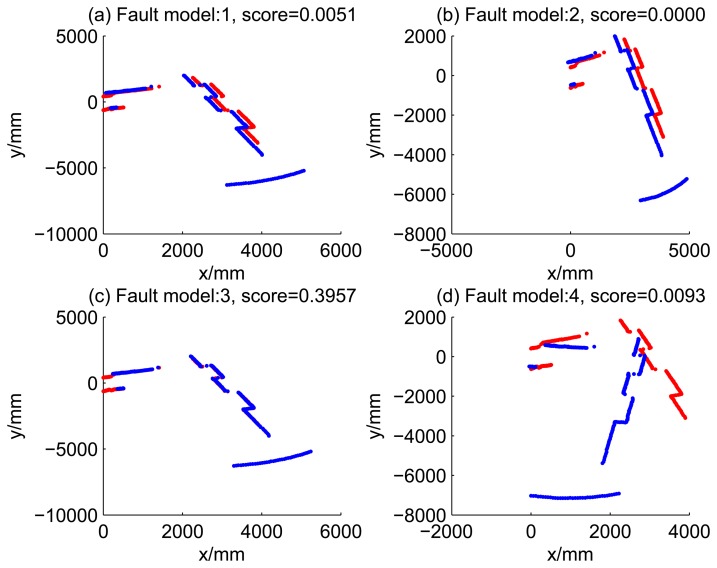
Case 3a: Right encoder stuck, fault model = 3 (time Step 10).

**Figure 18.**
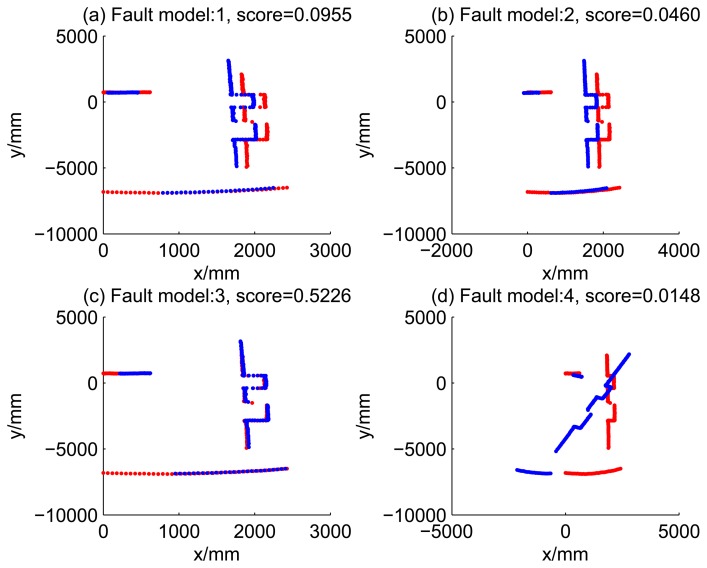
Case 3b: Right encoder stuck, fault model = 3 (time Step 13).

**Figure 19.**
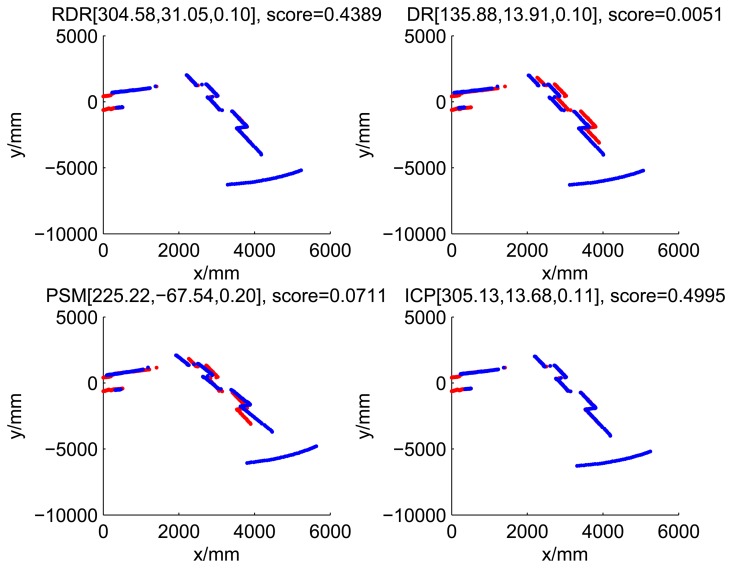
Robust dead reckoning result for Case 3a (time Step 10).

**Figure 20.**
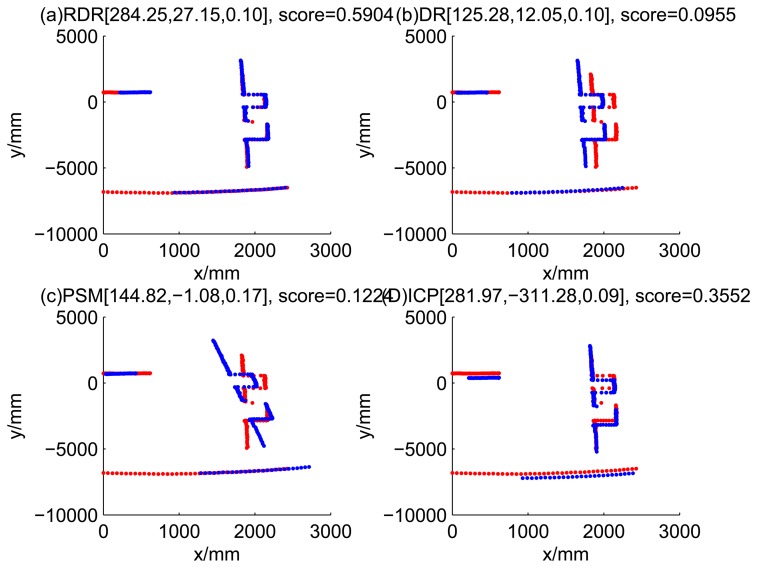
Robust dead reckoning result for Case 3b (time Step 13).

**Figure 21.**
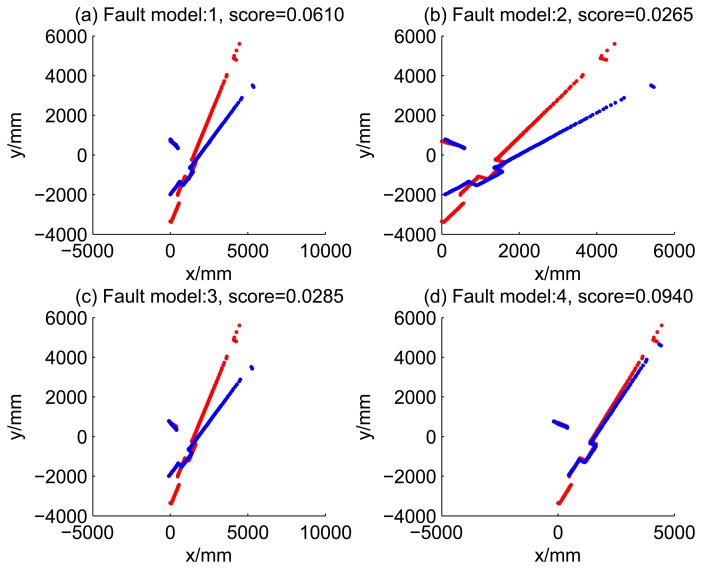
Case 4a: Gyroscope stuck, fault model = 4 (time Step 16).

**Figure 22.**
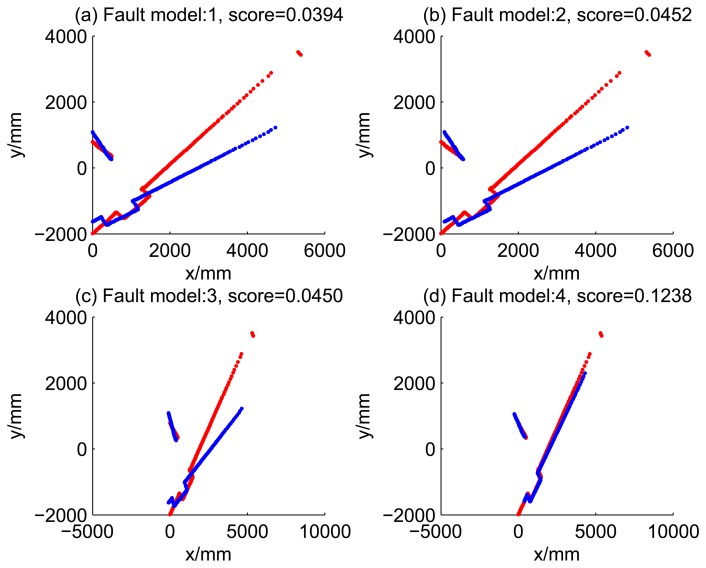
Case 4b: Gyroscope stuck, fault model = 4 (time Step 17).

**Figure 23.**
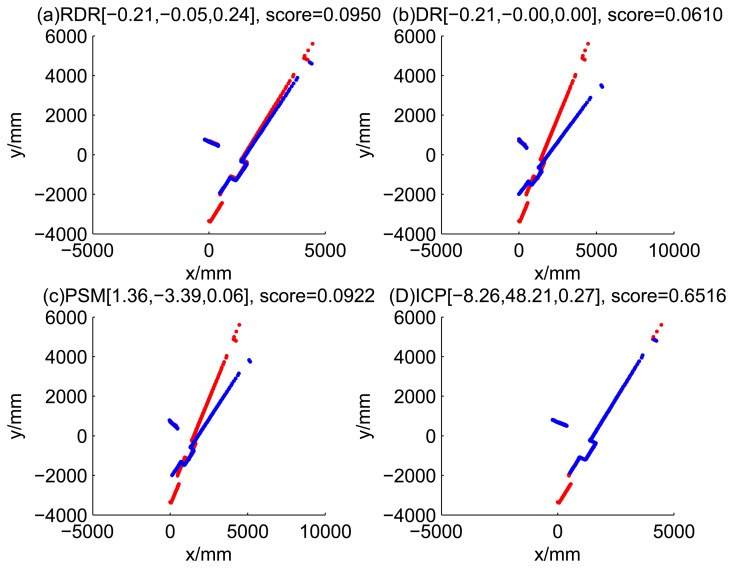
Robust dead reckoning result for Case 4a (time Step 16).

**Figure 24.**
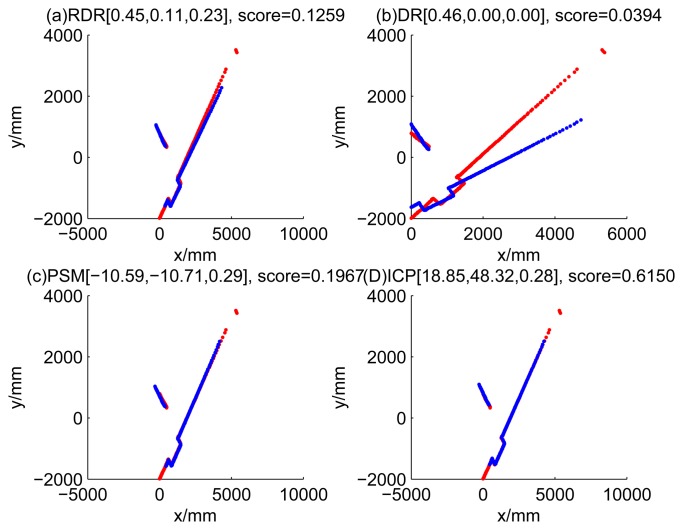
Robust dead reckoning result for Case 4b (time Step 17).

**Table 1.** Fault Models for Mobile Robots.

**Fault Models**	**Fault Components**	**Kinematics**
1	(No Faults)	Equation (19)
2	Left Encoder	Equation (21)
3	Right Encoder	Equation (22)
4	Gyroscope	Equation (23)

**Table 2.** Likelihood of dead reckoning for four models.

**t**	**Model 1**	**Model 2**	**Model 3**	**Model 4**
1	0.3103	0.2485	0.4062	0.1247
2	0.1538	0.2021	0.1218	0.1598
3	0.5538	0.6027	0.4523	0.2907
4	0.1708	0.2006	0.1200	0.1690
5	0.0626	0.2583	0.0370	0.1165
6	0.0053	0.6390	0.0216	0.0301
7	0.0025	0.5691	0.0043	0.0346
8	0.0215	0.3831	0.0088	0.0378
9	0.0145	0.2756	0.0040	0.0081
10	0.0051	0.0000	0.3957	0.0093
11	0.0159	0.0009	0.5132	0.0105
12	0.1272	0.1056	0.1986	0.0151
13	0.0955	0.0460	0.5226	0.0148
14	0.0327	0.0040	0.2864	0.0103
15	0.0923	0.0852	0.0555	0.2845
16	0.0610	0.0265	0.0285	0.0940
17	0.0394	0.0452	0.0450	0.1238
18	0.0648	0.0207	0.0726	0.2077
19	0.0355	0.0158	0.0774	0.0949
20	0.1774	0.1013	0.1383	0.0353

## Appendix: Notation

The main notations and their meanings in the paper are listed in Table A1.

**Table A1.** Notations and meanings.

**Notation**	**Meanings**
**S**	set of discrete models
*t*	time step
*s_t_*	discrete model at *t*
x*_t_*	continuous state at *t*
{**z***_t_*}	measurements at *t*
$\left(s_{t}^{\left[i\right]},{\text{x}}_{t}^{\left[i\right]}\right)$	the *^i^th* particle
*N*	number of particles
$w_{t}^{\left[i\right]}$	weight of the *^i^th* particle
*R_t_*	range data at *t*
$b_{t}^{j}$	the *j* – *th* ray of scan *R_t_*
*L*	number of rays of scan
$\rho_{t}^{j}$	the range readings of $b_{t}^{j}$
$\alpha_{t}^{j}$	the orientation of $b_{t}^{j}$
*ϱ*	angular offset of the first ray
*ς*	angular resolution
$G_{t}^{k}$	the *k – th* segment of *R_t_*
*c_t_*	number of segments of *R_t_*
${\text{start}}_{t}^{k}$	start index of $G_{t}^{k}$
${\text{end}}_{t}^{k}$	end index of $G_{t}^{k}$
${\text{num}}_{t}^{k}$	numbers of rays in $G_{t}^{k}$
${\tilde{\rho }}_{t}^{k}$	average length of rays in $G_{t}^{k}$
*W_t_*	effective scan of *R_t_*
*A_t_*	homogeneous transformation matrix
*H_t_*	rotation matrix of *A_t_*
*P_t_*	translation vector of *A_t_*
*x_t_*	first dimension of **x***_t_*
*y_t_*	second dimension of **x***_t_*
*θ_t_*	third dimension of **x***_t_*
$d_{t}^{i}$	length of the projected ray of $b_{t}^{i}$
$\gamma_{t}^{i}$	orientation of the projected ray of $b_{t}^{i}$
$e_{t}^{i}$	discrepancy of ray $b_{t}^{i}$
*υ_t_*	linear speed at *t*
*θ̇_t_*	yaw rate at *t*
$\upsilon_{t}^{j}$	linear speed at *t* of model j
${\dot{\theta }}_{t}^{j}$	yaw rate at *t* of model j
$e_{t}^{L}$	linear velocity of left wheel obtained from left encoder
$e_{t}^{R}$	linear velocity of right wheel obtained from right encoder
*g_t_*	readings of gyroscope
*τ_t_*	interval of time step *t*
σ	deviation of range scan readings
